# ChatGPT’s Performance in Cardiac Arrest and Bradycardia Simulations Using the American Heart Association's Advanced Cardiovascular Life Support Guidelines: Exploratory Study

**DOI:** 10.2196/55037

**Published:** 2024-04-22

**Authors:** Cecilia Pham, Romi Govender, Salik Tehami, Summer Chavez, Omolola E Adepoju, Winston Liaw

**Affiliations:** 1 Tilman J Fertitta Family College of Medicine University of Houston Houston, TX United States; 2 Humana Integrated Health Sciences Institute University of Houston Houston, TX United States; 3 Department of Health Systems and Population Health Sciences Tilman J Fertitta Family College of Medicine Houston, TX United States

**Keywords:** ChatGPT, artificial intelligence, AI, large language model, LLM, cardiac arrest, bradycardia, simulation, advanced cardiovascular life support, ACLS, bradycardia simulations, America, American, heart association, cardiac, life support, exploratory study, heart, heart attack, clinical decision support, diagnostics, algorithms

## Abstract

**Background:**

ChatGPT is the most advanced large language model to date, with prior iterations having passed medical licensing examinations, providing clinical decision support, and improved diagnostics. Although limited, past studies of ChatGPT’s performance found that artificial intelligence could pass the American Heart Association’s advanced cardiovascular life support (ACLS) examinations with modifications. ChatGPT’s accuracy has not been studied in more complex clinical scenarios. As heart disease and cardiac arrest remain leading causes of morbidity and mortality in the United States, finding technologies that help increase adherence to ACLS algorithms, which improves survival outcomes, is critical.

**Objective:**

This study aims to examine the accuracy of ChatGPT in following ACLS guidelines for bradycardia and cardiac arrest.

**Methods:**

We evaluated the accuracy of ChatGPT’s responses to 2 simulations based on the 2020 American Heart Association ACLS guidelines with 3 primary outcomes of interest: the mean individual step accuracy, the accuracy score per simulation attempt, and the accuracy score for each algorithm. For each simulation step, ChatGPT was scored for correctness (1 point) or incorrectness (0 points). Each simulation was conducted 20 times.

**Results:**

ChatGPT’s median accuracy for each step was 85% (IQR 40%-100%) for cardiac arrest and 30% (IQR 13%-81%) for bradycardia. ChatGPT’s median accuracy over 20 simulation attempts for cardiac arrest was 69% (IQR 67%-74%) and for bradycardia was 42% (IQR 33%-50%). We found that ChatGPT’s outputs varied despite consistent input, the same actions were persistently missed, repetitive overemphasis hindered guidance, and erroneous medication information was presented.

**Conclusions:**

This study highlights the need for consistent and reliable guidance to prevent potential medical errors and optimize the application of ChatGPT to enhance its reliability and effectiveness in clinical practice.

## Introduction

In March 2023, OpenAI, an artificial intelligence (AI) research laboratory, released GPT-4, an experimental version of ChatGPT, a large language model chatbot [1]. Compared with prior models including its predecessor GPT-3.5, GPT-4 has higher accuracy, greater reasoning capabilities, superior confidence, and more safety features with company data demonstrating improved performance on the Uniform Bar Exam and Biology Olympiad [2,3]. Compared with other types of learning models, deep learning models use multiple processing layers and artificial neural networks to process large amounts of data in order for the AI to learn [4]. In health care, ChatGPT shows promising applications including passing the United States Medical Licensing Examination, individualized health advice, improved diagnostic care, faster pharmacologic discovery, task automation, and clinical decision support [5-10]. However, limited evidence exists regarding ChatGPT’s ability to perform in advanced clinical scenarios, including cardiac emergencies.

Currently, the standard of care follows algorithms from the 2020 American Heart Association (AHA) advanced cardiovascular life support (ACLS) guidelines. Learners enrolled in the AHA course take a multiple-choice cognitive examination followed by a series of scenario-based patient cases and skills demonstrations [11]. Although an early study by Fijačko et al [12] concluded ChatGPT was unable to pass the AHA’s basic life support and advanced cardiovascular life support (ACLS) examinations, they demonstrated ChatGPT was able to provide relevant and accurate explanations for scenario-based questions without learning from an AHA course. With modification, Zhu et al [13] determined ChatGPT was able to pass the AHA’s examinations after changing the research protocol in which 3 responses were generated for each question instead of 1, and assessments were turned into open-ended questions. While rudimentary tests find ChatGPT can quickly give well-tailored answers to questions and scenarios regarding cardiopulmonary resuscitation (CPR) based on published guidelines, this has not been rigorously applied to more complex clinical scenarios requiring higher levels of expertise and training [8].

Even with advances in modern health care, heart disease has remained the leading cause of death for several decades [14]. Approximately 436,000 Americans die from cardiac arrest each year, making it a significant public health issue [15]. Past efforts at reducing cardiac arrests include establishing cardiac arrest registries, increasing public awareness and educational efforts, improving the quality of resuscitation care, strengthening emergency medical services, quality improvement programs, and implementing accreditation standards [16]. ACLS protocols have been well-proven to improve cardiac arrest outcomes, with deviations from the guidelines associated with poorer survival and neurologic outcomes [17-22]. Thus, identifying technologies that can improve adherence to ACLS protocols and in turn, cardiac arrest outcomes, is critical. Cognitive aids, such as pocket cards, mobile apps, and augmented reality glasses, have been shown to increase adherence to ACLS guidelines and the quality of resuscitation in adult simulations for in-hospital cardiac arrests [23-28]. ChatGPT could prove to be a valuable resource by providing real-time decision support in resource-limited settings or augmenting clinical decision-making. Our study aims to analyze the accuracy of ChatGPT’s performance in following the AHA’s ACLS algorithms in 2 cardiac event simulations—cardiac arrest and bradycardia.

## Methods

We evaluated the accuracy of ChatGPT’s responses to 2 simulations based on the 2020 AHA ACLS guidelines with 3 primary outcomes of interest as follows: the mean individual step accuracy, the accuracy score per simulation attempt, and the accuracy score for each algorithm.

### Ethical Considerations

In accordance with 45 CFR 46, Subpart A, also known as the Common Rule, our research project involving educational tests, such as ACLS simulations, falls under the exemption outlined in 46.104(d)(1). This exemption applies because the interactions in our study are limited to educational tests and do not involve identifiable human subjects. Because of this, no ethics board review was required for this research.

### Initial Observations of ChatGPT’s Responses and Adherence to AHA Guidelines

The research team underwent an initial testing phase involving several procedures to observe ChatGPT’s responses before developing the scripts used for testing. Initially, two types of trials were proposed: (1) assessing ChatGPT’s ability to accurately identify rhythm strips, and (2) evaluating ChatGPT’s capability to execute the algorithm provided by the AHA. However, from January to May 2023, ChatGPT lacked the ability to recognize images, including rhythm strips or electrocardiograms (ECGs). Due to this limitation, specific ECG rhythms were provided to ChatGPT without the need for correct identification. The team then tested ChatGPT’s ability to execute basic algorithms, including those for bradycardia, tachycardia with a pulse, and cardiac arrest, based on the 2020 AHA Guidelines. At the beginning of the trials, it was confirmed by ChatGPT that it would refer to the 2020 AHA Guidelines for information. During these initial trials, testers observed significant differences between the responses generated by GPT-3.5 and GPT-4. GPT-4 demonstrated greater adherence to the 2020 AHA Guidelines by being able to interpret the algorithmic pathways more accurately. Additionally, testers noted that prompting ChatGPT led to lengthy outputs, unlike the step-by-step approach used in real-life simulations. To emulate real-life testing conditions in ACLS training, the command “What is the next step? Give me one step at a time” was included. This method also mimics how ChatGPT could be used in the real world. As GPT-4 closely followed the 2020 AHA Guidelines and was able to respond to the step-by-step command, it was selected for the actual simulations.

### Development of Scripts for User Input and ChatGPT Output

Following the completion of the initial trials, the study authors with prior ACLS training (SC, WL, and CP) developed scripts based on the testers’ observations, the AHA’s algorithms, and clinical experience. SC and WL are both physicians in emergency medicine and family medicine, respectively, while CP holds an EMT license with paramedic-level training. The 2 scripts exclusively focused on testing 2 categories of cardiac arrhythmias: bradycardia and cardiac arrest (Multimedia Appendices 1 and 2). To validate the scripts, 3 physicians with ACLS and emergency medicine backgrounds assessed them to ensure they accurately depict real-world clinical scenarios and management practices. The study authors developed a scoring guide to measure the accuracy of the responses of ChatGPT to prespecified prompts. The number of attempts given to ChatGPT was matched to the required correct outputs for each group. For instance, if 5 correct outputs were expected, ChatGPT was prompted 5 times, allowing it the same number of opportunities to provide accurate responses. In instances where ChatGPT’s outputs diverged from the predefined script, the authors responded by typing “Give me one step at a time” or “Give me another option.”

### Data Collection

Testing was conducted between May to August 2023 using the public web user chat interface by OpenAI, with upgraded accounts to use GPT-4. All inputs and outputs for 1 simulation are saved within a single conversation thread, and each simulation was conducted in a separate thread. Three testers underwent training to adhere to the scripts, ensuring standardized procedures for the simulations. Each scenario was tested 20 times to evaluate the variability of ChatGPT’s responses and its ability to provide correct outputs. For each simulation step, ChatGPT was scored for correctness (1 point) or incorrectness (0 points), and these scores were recorded in an Excel (Microsoft Corp) spreadsheet. Partially correct responses were considered incorrect. Within each scoring section, qualitative comments regarding the incorrect responses were also recorded. Additionally, instances where the prompt “Give me one step at a time” requiring repeated inputs were recorded. The full transcripts for each simulation were collated into separate document files for each simulation attempt. The authors of the scripts independently reviewed the recorded accuracy scores in the spreadsheets and the transcripts saved in the document files to ensure the validity of the results and to maintain accurate record-keeping.

### Data Analysis

Using the data recorded in the Excel spreadsheets, accuracy scores for individual steps, individual simulation attempts, and overall simulation attempts for each algorithm were calculated. The overall score per simulation attempt was calculated by summing the correct responses and dividing them by the number of steps (12 for bradycardia and 39 for cardiac arrest). For each step in the simulation, we similarly summed the correct responses and divided them by the number of simulations (n=20). Mean overall simulation accuracies were determined by averaging 20 simulation scores. The median accuracies for each algorithm were also calculated. Qualitative comments recorded for each simulation were also analyzed to identify recurring themes to describe ChatGPT’s performance.

## Results

### Overview

The individual step accuracy per simulation attempt for the cardiac arrest and bradycardia algorithms are reported in Tables 1 and 2, respectively. ChatGPT’s median accuracy for each step was 85% (IQR 40%-100%) for cardiac arrest and 30% (IQR 13%-81%) for bradycardia. The accuracy scores per simulation attempt for each algorithm are described in Table 3. ChatGPT’s median accuracy for over 20 simulation attempts for cardiac arrest was 69% (IQR 67%-74%) and for bradycardia was 42% (IQR 33%-50%). Four key findings were identified after analyzing the results from the 2 sets of simulations: (1) ChatGPT’s outputs varied despite consistent input, (2) the same actions were persistently missed, (3) repetitive overemphasis hindered guidance, and (4) erroneous medication information was presented.

**Table 1:** Individual step accuracy using ChatGPT in cardiac arrest advanced cardiovascular life support simulations (N=20).

Correct simulation output			Accuracy, n (%)^a^
**Part 1: Initiation of CPR^b^**			
	Assess for responsiveness	20 (100)	
	Assess for a pulse	20 (100)	
	Activate an emergency response system	19 (95)	
	Start CPR	20 (100)	
	Apply the defibrillator	20 (100)	
	Obtain intravenous or intraosseous access	2 (10)	
**Part 2: First dose of epinephrine**			
	Assess for responsiveness	0 (0)	
	Assess for a pulse	4 (20)	
	Continue CPR	17 (85)	
	Give 1 mg of epinephrine	19 (95)	
	Consider advanced airway management	13 (65)	
	“Continue CPR” after completing Part 2	16 (80)	
**Part 3: First rhythm check**			
	Perform a rhythm check	20 (100)	
	Consider reversible causes	8 (40)	
	Continue CPR	20 (100)	
**Part 4: Second dose of epinephrine**			
	Perform a rhythm check	12 (60)	
	Immediately defibrillate	11 (55)	
	Give 1 mg of epinephrine	15 (75)	
	Consider reversible causes	4 (20)	
	“Continue CPR” after completing Part 4	20 (100)	
**Part 5: Second defibrillation**			
	Perform a rhythm check	18 (90)	
	Immediately defibrillate	19 (95)	
	“Continue CPR” after completing Part 5	20 (100)	
**Part 6: First dose of alternative pharmacologic agents**			
	Give 1 mg of epinephrine	6 (30)	
	Consider giving amiodarone or lidocaine	9 (45)	
	Consider reversible causes	0 (0)	
	Perform a rhythm check	20 (100)	
	Immediately defibrillate	20 (100)	
	“Continue CPR” after completing Part 6	16 (80)	
**Part 7: Additional pharmacologic agents**			
	Perform a rhythm check	19 (95)	
	Immediately defibrillate	18 (90)	
	Consider reversible causes	2 (10)	
	Give a second dose of amiodarone	6 (30)	
	“Continue CPR” after completing Part 7	19 (95)	
**Part 8: End of simulation**			
	Give 1 mg of epinephrine	14 (70)	
	Consider giving lidocaine	0 (0)	
	Consider reversible causes	11 (55)	
	Perform a rhythm check	18 (90)	
	“Continue CPR” after completing Part 8	20 (100)	

^a^Median accuracy is 85% (IQR 40%-100%); mean accuracy is 69%.

^b^CPR: cardiopulmonary resuscitation.

**Table 2:** Individual step accuracy using ChatGPT in bradycardia advanced cardiovascular life support simulations (N=20). Mean individual step accuracy using ChatGPT in bradycardia advanced cardiovascular life support simulations (N=20).

Correct simulation output			Accuracy, n (%)^a^
**Part 1: Address ABC’s of resuscitation**			
	Maintain the patient’s airway; assists breathing as necessary	16 (80)	
	Apply a cardiac monitor to identify rhythm	3 (15)	
	Monitor blood pressure, pulse oximetry, and other vital signs	9 (45)	
	Obtain intravenous access	7 (35)	
	Obtain 12-lead ECG^b^	0 (0)	
	Give 1 mg of atropine	0 (0)	
**Part 2: Address hypoxia**			
	Give oxygen	20 (100)	
**Part 3: Second dose of atropine**			
	Give an additional dose of atropine	19 (95)	
**Part 4: Alternative pharmacologic agents and consideration of pacing**			
	Give an additional dose of atropine	3 (15)	
	Consider transcutaneous pacing	17 (85)	
	Give dopamine or epinephrine	1 (5)	
**Part 5: Seek expert consultation**			
	Seek a consultation	5 (25)	

^a^Median accuracy is 30% (IQR 13%-81.3%); mean accuracy is 42%.

^b^ECG: electrocardiogram.

**Table 3:** Accuracy score per simulation attempt for ChatGPT in cardiac arrest (N=39) and bradycardia (N=12) advanced cardiovascular life support simulations.

Simulation number	Accuracy of cardiac arrest, n (%)^a^	Accuracy of bradycardia, n (%)^b^
1	24 (62)	7 (58)
2	26 (67)	5 (42)
3	24 (62)	6 (50)
4	29 (74)	6 (50)
5	27 (69)	5 (42)
6	27 (69)	7 (58)
7	21 (54)	4 (33)
8	29 (74)	4 (33
9	26 (67)	7 (58)
10	27 (69)	6 (50)
11	26 (67)	5 (42)
12	26 (67)	5 (42)
13	22 (56)	5 (42)
14	26 (67)	5 (42)
15	27 (69)	3 (25)
16	29 (74)	7 (58)
17	29 (74)	3 (25)
18	30 (77)	5 (42)
19	30 (77)	3 (25)
20	30 (77)	4 (33)
Average accuracy	535/780 (69)	102/240 (43)

^a^Median accuracy of cardiac arrest is 69% (IQR 67%-74%).

^b^Median accuracy of bradycardia is 42% (IQR 33%-50%).

### ChatGPT’s Outputs Varied

ChatGPT gave varying responses to identical inputs, sometimes providing unexpected responses. ChatGPT generated multistep instructions, prompting the request “give me one step at a time.” It would sometimes provide entirely different steps that were not in its original multistep output. There were, on average, 0.5 instances per simulation of repetitive loops of “check heart rhythm” and “resume CPR.” Even when “one step at a time” was requested, ChatGPT was unable to consistently execute algorithms step by step, often generating multistep instructions. ChatGPT was instructed to give “one step at a time” 3.6 times per simulation in the cardiac arrest case and 1.5 times per simulation in the bradycardia case. Other unexpected outputs include 0.5 instances per simulation of oxygen and medication delivery instructions preceding necessary steps such as applying a monitor to the patient or starting an intravenous (IV) line. ChatGPT provided additional instructions that were not part of the ACLS algorithms, including 0.3 instances per simulation of “start an isoproterenol infusion.”

### Some Actions Were Persistently Missed

Throughout the simulations, there were several notable omissions to ChatGPT’s output. ChatGPT frequently skipped the instruction to establish IV or intraosseous (IO) access, before directing IV or IO medication administration at 0% accuracy in cardiac arrest and 35% (n=7) accuracy in bradycardia. ChatGPT also struggled to incorporate additional dosages of medications in its responses. For epinephrine administration, ChatGPT was accurate 95% (n=19) of the time regarding the first dose, but its accuracy decreased to 75% (n=15) and 70% (n=14) for the second and third doses, respectively. In the bradycardia simulations, ChatGPT consistently recommended a second dose of atropine at 95% (n=19) accuracy but rarely advised a third dose at 15% (n=3) accuracy. Furthermore, ChatGPT was never prompted to obtain a 12-lead ECG with 0% accuracy. ChatGPT did not provide any instructions for checking vital signs and only suggested pulse checks for the patient with bradycardia, omitting other vital signs such as blood pressure and oxygen saturation.

### Repetitive Overemphasis Hindered Guidance

ChatGPT consistently overemphasized specific steps to the detriment of other important interventions. During the cardiac arrest simulations, ChatGPT became fixed in repetitive cycles when given the prompt, “CPR resumed. It’s been two minutes. What is the next immediate step?” ChatGPT would then incessantly advise to “check the heart rhythm” and “resume CPR” on average 4 times per simulation without providing any other instructions such as considering advanced airway management at 65% (n=13) accuracy and exploring reversible causes with 25% (n=5) accuracy. Similarly, in the bradycardia simulation, ChatGPT emphasized transcutaneous and transvenous pacing methods on average 1.8 times per simulation, disregarding other reasonable alternatives for bradycardia management, such as pharmacologic interventions.

### Erroneous Medication Information Was Presented

Two medication instructions given in the bradycardia simulations were consistently inaccurate. In all bradycardia simulations, ChatGPT advised administering 0.5 mg of atropine instead of the recommended 1 mg dose in the most recent 2020 AHA ACLS guidelines (0% accuracy). Despite being corrected and acknowledging the accurate dosage as 1 mg, ChatGPT persisted in administering the incorrect dose of atropine in subsequent outputs. In 95% (n=19) of bradycardia simulations, ChatGPT provided incorrect guidance to administer 2-20 µg/kg/minute of dopamine, deviating from the correct range of 5-20 µg/kg/minute. However, in all cardiac arrest simulations, all medication dosages were correctly identified by ChatGPT.

## Discussion

### Principal Findings

This study sought to examine the accuracy of ChatGPT’s performance in adult cardiac event simulations for cardiac arrest and bradycardia. Although the overall accuracy of ChatGPT was lower than the standard passing threshold in academics of 70%, the demonstrated accuracy of ChatGPT was comparable to one other study of complex cardiac clinical vignettes which had 50% accuracy (50/100). Compared with experts, ChatGPT gave inaccurate or incomplete responses [29]. Even with these caveats, this proof-of-concept study illustrates the potential and perils of using ChatGPT for real-time decision support in clinical settings.

Our study adds to the small but growing body of literature analyzing the potential clinical role of ChatGPT and other AI technologies, with a special focus on cardiac emergencies and complex decision-making. While this field may be in its infancy, ChatGPT builds upon prior generations of more low-tech cognitive aids such as apps or paper cards by offering faster responses, tailored answers, and the ability to work through far more complicated scenarios. In several steps of the simulations, ChatGPT’s accuracy was as high as 100% (n=20), an impressive benchmark.

In practice settings with limited resources, ChatGPT, AI, and related technologies may still help fill a crucial gap by acting as a more advanced cognitive aid compared with previously studied solutions. They can also be used in medical education, quality initiatives, and simulation training exercises. However, the findings from this study highlight several key issues that need to be addressed before implementing this decision aid in patient care.

In our study, ChatGPT exhibited a wide range of clinical accuracy, which can be due in part to outdated or unreliable training data. If AI is used to augment clinical decision-making, physicians must ensure the appropriateness and validity of the training data. Without this level of accountability, health care professionals could accept hallucinations, or AI responses that are incorrect or misleading. While subject matter experts may detect these mistakes, those with less training and experience may fail to recognize these inconsistencies, which can lead to missing steps or delivering inaccurate medication doses. Additionally, if the training data contains inaccuracies or reflects certain biases, ChatGPT may replicate those errors or biases in its responses. It is also unclear how frequently ChatGPT updates or how it decides to include new or controversial scientific findings in its model. This is especially salient in cardiac emergencies, where quick and accurate decision-making is crucial for patient survival.

Our findings suggest that ChatGPT has difficulty learning from past scenarios. For example, ChatGPT would acknowledge the correct dosage if it initially gave an incorrect one, but persistently administered the same incorrect doses across subsequent trials. Real-life ACLS scenarios involve complex medical situations with multiple possible interventions depending on the patient’s status. Patients with cardiac arrest secondary to hyperkalemia or pulmonary embolism are managed differently than the simpler cases created for this simulation. Achieving higher levels of accuracy would either require the ability for ChatGPT to learn or a different AI model altogether.

Some experts recommend asking probing questions to AI as a possible solution to verify responses from ChatGPT. In clinical scenarios where time directly correlates with myocardial health and patient outcomes, this may be unrealistic. Continuing to prompt the AI or slightly modifying responses to see how responses change is also not practical. In our study, ChatGPT was prompted dozens of times for responses. During an actual episode of cardiac arrest, where intervals of CPR are done in 2 minutes, spending most of the time repeatedly prompting ChatGPT for an accurate response is probably not time well spent during the resuscitation.

Several limitations should be considered when interpreting these results. First, our study focused only on 2 ACLS scenarios: bradycardia and cardiac arrest. Because this is a proof-of-concept study, the authors decided to test just 2 scenarios. In the overall ACLS framework, there are many other emergencies covered, including the management of tachyarrhythmias, opioid overdose, and stroke. Given that ChatGPT’s performance could vary in these alternative scenarios, broadening the scope of simulations might provide a more thorough insight into ChatGPT’s capabilities by evaluating its performance across a broader range of ACLS conditions. Our study analyzed the accuracy of ChatGPT only, making it difficult to generalize the results to other large language models. During the study period, ChatGPT could not recognize images, requiring the study authors to specify the cardiac rhythm in the model, potentially affecting the accuracy. There are growing concerns that the quality of ChatGPT has decreased over time as it has become more widely used by the public, which could also impact the results [30]. Additionally, given the potential evolution of ChatGPT’s capabilities over time, the reliability and validity of the study’s results may be affected. Exploring how AI models can update their knowledge could provide insights into their long-term utility in real-world medical emergency settings.

### Conclusions

Bridging the gap between AI and human language is a multifaceted challenge, balancing advancements, data quality, and human oversight to maximize ChatGPT’s benefits and minimize risks in health care. This study highlights the need for consistent and reliable guidance to prevent potential medical errors and optimize the application of ChatGPT to enhance its reliability and effectiveness in clinical practice. To enhance ChatGPT’s real-world effectiveness, significant improvements are needed, particularly in accuracy and consistency across diverse medical situations. While ChatGPT holds promise as a decision support tool that can provide structured clinical guidance, it should complement, not replace, qualified health care professionals. Future research also should aim to address these limitations and further investigate the challenges of AI in health care to ensure its safe and effective use. Additionally, future studies comparing ChatGPT’s performance with other AI models or with human performance could offer valuable insights.

## Appendix

Multimedia Appendix 1 ChatGPT ACLS Testing Algorithm for Bradycardia.

Multimedia Appendix 2 ChatGPT ACLS Testing Algorithm for Cardiac Arrest.

## Abbreviations

ACLS: advanced cardiovascular life support
AHA: American Heart Association
AI: artificial intelligence
CPR: cardiopulmonary resuscitation
ECG: electrocardiogram
IO: intraosseous
IV: intravenous

## Data Availability

The data sets generated during or analyzed during this study are available from the corresponding author upon reasonable request.
