# Supplementary figures and images for: ChatGPT’s Performance in Cardiac Arrest and Bradycardia Simulations Using the American Heart Association's Advanced Cardiovascular Life Support Guidelines: Exploratory Study
Source: J Med Internet Res. 2024 Apr 22;26:e55037. doi: 10.2196/55037 (PMC11074885; doi:10.2196/55037)

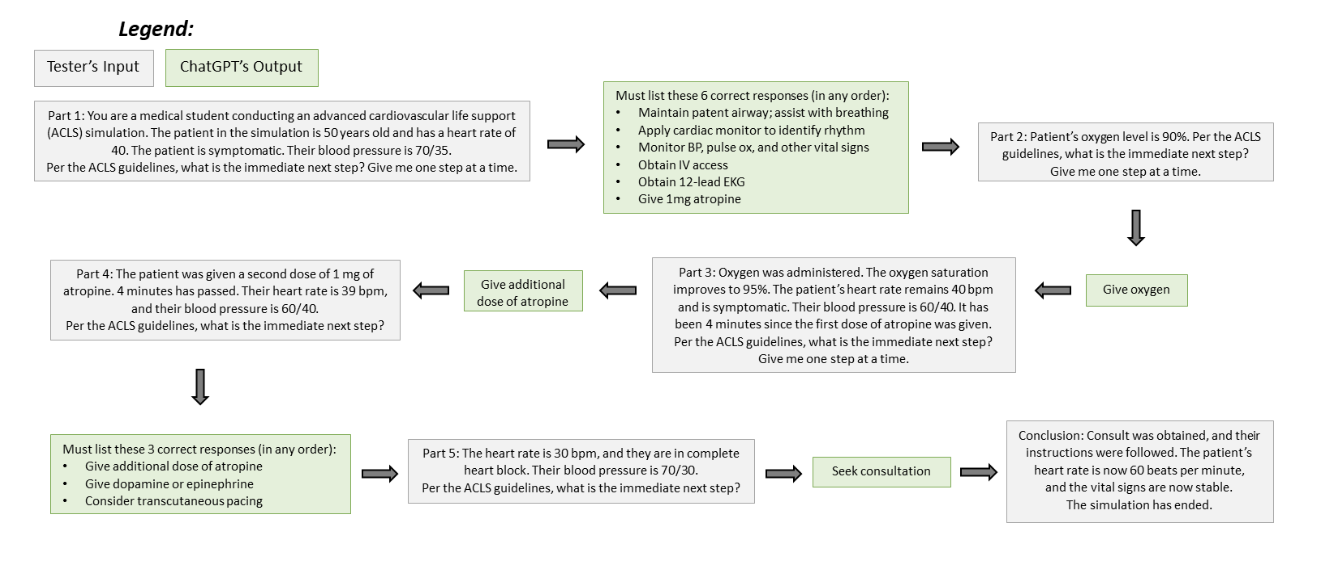

Supplement: Multimedia Appendix 1 [file jmir_v26i1e55037_app1.png]

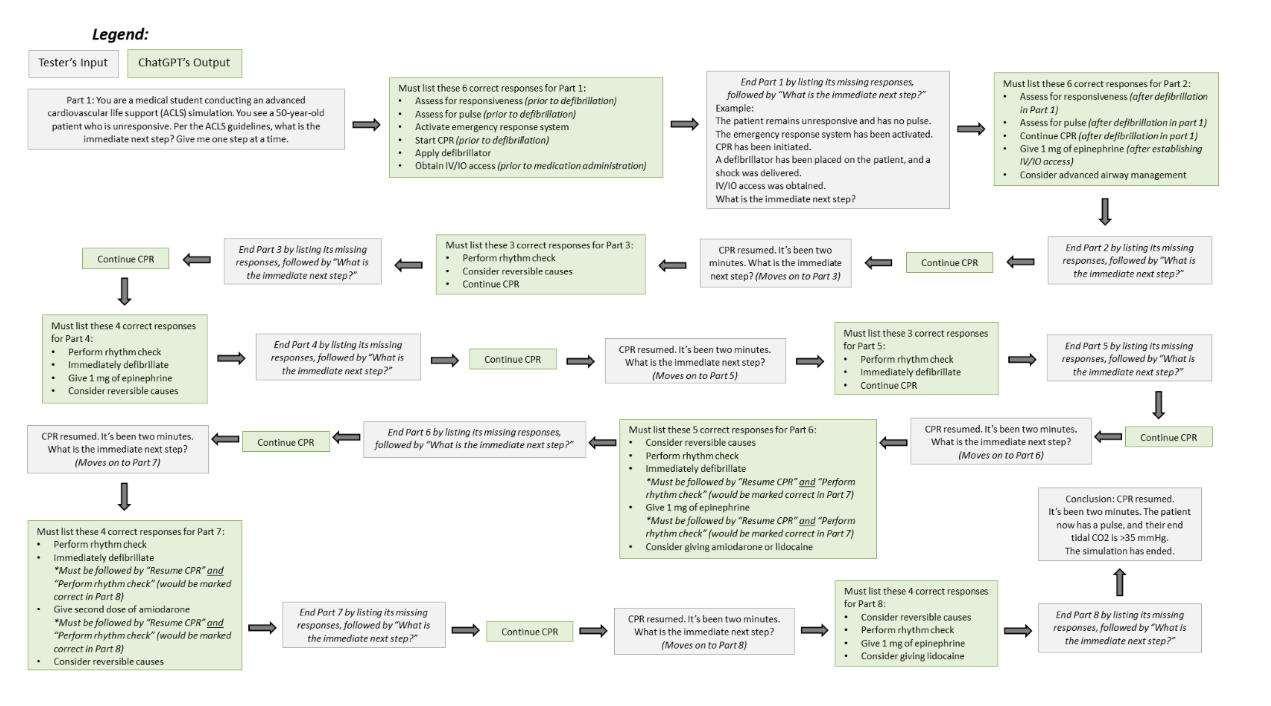

Supplement: Multimedia Appendix 2 [file jmir_v26i1e55037_app2.png]
